# *ATRX* mutations mediate an immunogenic phenotype and macrophage infiltration in neuroblastoma

**DOI:** 10.1016/j.canlet.2025.217495

**Published:** 2025-01-30

**Authors:** Federica Lorenzi, Sina Jostes, Qiong Gao, J. Ciaran Hutchinson, Jennifer Tall, Barbara Martins da Costa, Anisha J. Cooke, Dyanne Rampling, Olumide Ogunbiyi, Karen Barker, Debbie Hughes, Giuseppe Barone, Marta Barisa, Angela Bellini, Michael Hubank, Gudrun Schleiermacher, John Anderson, Emily Bernstein, Louis Chesler, Sally L. George

**Affiliations:** a Division of Clinical Studies, The Institute of Cancer Research, 15 Cotswold Road, Sutton, SM2 5NG, London, United Kingdom; b Department of Oncological Sciences, Tisch Cancer Institute, Icahn School of Medicine at Mount Sinai, 1 Gustave L. Levy Place, New York, 10029-5674, USA; c Cancer Therapeutics Unit, Computational Biology and Chemogenomics, The Institute of Cancer Research, 15 Cotswold Road, Sutton, SM2 5NG, London, United Kingdom; d Histopathology Department, Great Ormond Street Institute of Child Health, Great Ormond Street, London, WC1N 3JH, United Kingdom; e UCL Great Ormond Street Institute of Child Health, Great Ormond Street, London, WC1N 3JH, United Kingdom; f SiRIC RTOP (Recherche Translationelle en Oncologie Pediatrique), U830 INSERM and SIREDO Integrated Pediatric Oncology Center, Institut Curie, 26 Rue d’Ulm, 75005, Paris, France; g Molecular Pathology Section, The Institute of Cancer Research, Clinical Genomics, The Royal Marsden NHS Foundation, 15 Cotswold Road, Sutton, SM2 5NG, London, United Kingdom; h Children and Young People’s Unit, The Royal Marsden Hospital, Downs Road, Sutton, SM2 5PT, London, United Kingdom

**Keywords:** Neuroblastoma, ATRX, Macrophages, Tumour microenvironment

## Abstract

*ATRX* is one of the most frequently mutated genes in high-risk neuroblastoma. *ATRX* mutations are mutually exclusive with *MYCN* amplification and mark a recognizable patient subgroup, presenting in older children with chemotherapy-resistant, slowly progressive disease. The mechanisms underlying how *ATRX* mutations drive high-risk and difficult-to-treat neuroblastoma are still largely elusive.

To unravel the role of ATRX in neuroblastoma, we generated isogenic neuroblastoma cell line models with *ATRX* loss-of-function and *ATRX* in-frame multi-exon deletions, representing different types of alterations found in patients. RNA-sequencing analysis consistently showed significant upregulation of inflammatory response pathways in the *ATRX*-altered cell lines. *In vivo*, *ATRX* alterations are consistently associated with macrophage infiltration across multiple xenograft models. Furthermore, *ATRX* alterations also result in upregulation of epithelial-to-mesenchymal transition pathways and a reduction in expression of adrenergic core-regulatory circuit genes.

Consistent with this, bioinformatic analysis of previously published neuroblastoma patient data sets revealed that *ATRX*-altered neuroblastomas display an immunogenic phenotype and higher score of macrophages (with no distinction between M1 and M2 macrophage populations) and dendritic cells, but not lymphocytes. Histopathological assessment of diagnostic samples from patients with *ATRX* mutant disease confirmed these findings with significantly more macrophage infiltration compared to *MYCN*-amplified tumors. In conclusion, we show that gene expression and cell-state changes as a result of *ATRX* alterations associate with a characteristic immune cell infiltration in both *in vivo* models and patient samples. Together, this provides novel insight into mechanisms underlying the distinct clinical phenotype seen in this group of patients.

## 1. Introduction

*ATRX* (alpha-thalassemia mental retardation X-linked) is a tumor suppressor gene, frequently mutated in various types of cancer, which encodes a SWI/SNF-like chromatin remodeler. Critical cellular functions of ATRX include maintenance of repetitive DNA regions, transcriptional repression, resolution of secondary DNA structures, protection of stalled replication forks, regulation of homologous recombination repair and the regulation of cellular senescence [1].

Neuroblastoma is a childhood tumor of the peripheral nervous system, which originates from abnormal development of sympathoadrenal progenitor cells [2]. Patients with high-risk neuroblastoma face only a 50 % chance of survival, despite intensive treatment [3]. In high-risk neuroblastoma, there are very few recurrently altered genes: *MYCN* amplification is the most frequent, detected in 35 % patients. *ATRX* alterations are detected in 10 % of patients and are mutually exclusive with *MYCN* amplification, thus defining a specific subgroup of high-risk patients [4,5].

Recent studies have shown that in neuroblastoma 68 % of *ATRX* mutations are multi-exon deletions, most commonly resulting in loss of exons 2–10 of the gene, and resulting in an in-frame fusion (IFF) protein with gain of function oncogenic effects [6,7]. The remainder of mutations are either nonsense or missense mutations that are thought to cause loss-of-function (LoF) of ATRX [7]. In all neuroblastoma cases, *ATRX* alterations are closely associated with alternative lengthening of telomeres (ALT), chronic metastatic disease and poor outcome [4,7,8]. Due to a lack of experimental models and limited understanding of ATRX biology, there is a scarcity of novel targeted therapies for children with *ATRX*-mutant neuroblastoma.

To meet this necessity, we generated isogenic models of *ATRX* LoF and IFF deletions by CRISPR-Cas9 technology. We identified an inflammatory phenotype characterized by an upregulation of inflammation-associated genes in cell line models, and an abundance of macrophages in *in vivo* models. Bioinformatic analysis of available patient data sets and histopathological assessment of diagnostic biopsies revealed an immunogenic microenvironment in *ATRX*-mutated tumors, consisting of a predominant contribution from the myeloid compartment. Our findings identify key differences in the tumor immune microenvironment in patients with *ATRX* mutant neuroblastoma that may, at least in part, explain the distinct clinical phenotype observed in these patients.

## 2. Methods

### 2.1. Cell culture, generation of isogenic cell lines by CRISPR-Cas9

Neuroblastoma NBL-S and SKNSH cell lines were purchased from ATCC and cultured in Dulbecco’s modified Eagle medium (Gibco, 11594446) with 10 % fetal bovine serum (Sigma-Aldrich, F2442). Cell lines were verified by STR profiling, routinely tested in the laboratory and approved as mycoplasma-free. NBL-S and SKNSH cells were transfected with Cas9-gRNA plasmids to generate *TP53* knockout and *ATRX* knockout as previously described [9]. For generation of *ATRX* 1–11 IFF, the *ATRX* wildtype *TP53* knockout NBL-S line A was transfected with two CRISPR gRNAs (Supplementary Table S1) encoded on pSpCas9 (BB)-2A-GFP (PX458) and pSpCas9(BB)-2A-Puro V2.0 (PX459), respectively, to induce a deletion of exons 2–10 (fusion of exon 1–11). Twenty-four hours post transfection cells were selected with puromycin (1 μg/ml) and sorted into a pool of GFP + cells. After 1–2 weeks, cells were sorted into single cells. DNA was collected from single cell clones and screened via PCR (for primers, Supplementary Table S2). IFF and wildtype clones were confirmed via targeted sequencing and Western blot for full-length ATRX or ATRX IFF.

### 2.2. C-circle assay and telomere quantitative PCR

Genomic DNA was isolated using DNeasy blood and tissue kit (Qiagen, 69504) following manufacturers’ instruction. As per published protocol [10], DNA was digested by 4 U/μg *Rsa*I and *Hin*fI and treated with RNAse for 1 h at room temperature (RT). C-circles were amplified by ϕ29 DNA polymerase, transferred by dotblot and detected by chemiluminescence after hybridization using TeloTAGG Telomere Length Assay Kit (Roche, 12209136001). In every blot, CHLA-90 (ALT-positive cells), no DNA sample and no ϕ29 polymerase sample per each cell line were added as controls. Quantification of dot intensity was performed by ImageJ and normalized to the intensity of corresponding samples ran without polymerase.

For telomere qPCR, as previously described [11], telomere and VAV (single copy gene) were quantified by SYBR green in two separate PCR reactions. Samples were loaded in triplicate and analyzed using the standard curve method. Telomere product was normalized to VAV.

### 2.3. Western blot and immunohistochemistry

Total protein was extracted by Pierce IP Lysis buffer (Thermo Scientific, 87788). Immunoblots were probed overnight with antibodies listed in Supplementary Table S3. For immunohistochemistry analysis, sections of 4 μm were dewaxed, rehydrated and washed with tap water. Antigen retrieval was achieved by immersing the sections into boiling citrate buffer (Sigma-Aldrich, 25114). Endogenous peroxidases were blocked with 1.6 % H_2_O_2_ for 10 min. Sections were blocked with 5 % bovine serum albumin for 30 min, then probed with primary antibody overnight in cold room (Supplementary Table S3). After washing in PBS, sections were incubated with secondary antibody for 45 min and incubated with avidin HRP-conjugated for 2 h. The chromogenic reaction was catalyzed by DAB substrate solution (Vector laboratories, SK-4105). Sections were counterstained, dehydrated and mounted. F4/80 staining intensity was calculated as a ratio between the DAB positively stained cells and total number of cells by ImageJ software. Staining intensity was measured in 4 views of 4 sections/mice per cell line.

### 2.4. Cytokine array

Cell supernatants of NBL-S *ATRX* knockout and wildtype lines were harvested 72 h after plating and analyzed by human cytokine array kit as per instructions (R&D systems, ARY005B). NBL-S *ATRX* IFF and wildtype cells were starved in DMEM 0.5 % FBS for 24 h and then medium was exchanged for standard DMEM 10 % FBS to stimulate cytokine production. Cell supernatants were harvested 48 h later and sent to Eve Technologies (Calgary, Canada) for cytokine array analysis. Cytokine levels were normalized to cell number at timepoint of harvest.

### 2.5. RNA extraction and sequencing

RNA was isolated via RNeasy Plus kit (Qiagen, 74134) according to manufacturer’s instructions. RNA sequencing of NBL-S *ATRX* knockout lines was carried out at the Genomics facility at the ICR. For RNA-seq of *ATRX* IFF models, RNA was isolated via RNeasy Mini kit (Qiagen, 74104) and quality of RNA samples was assessed on a 2100 Agilent Bioanalyzer. mRNA was extracted from 2 μg of total RNA per sample using NEXTFLEX^®^ Poly(A) Beads 2.0 (PerkinElmer). Libraries were prepared using NEXTFLEX^®^ Rapid Directional RNA-seq Kit 2.0 (PerkinElmer). Quality of library preparation was assessed on a 2100 Agilent Bioanalyzer or 4150 Tapestation (Agilent). Paired-end 60-bp reads were sequenced on the NextSeq2000 according to the manufacturer’s guidelines (Illumina).

### 2.6. RNA-sequencing analysis

For NBL-S *ATRX* knockout lines, RNA-seq paired-end reads (read length 100 base pairs) were aligned to the human GRCh38 reference genome and read counts for each gene were calculated with STAR Aligner (star/2.7.6a). The gene read counts were normalized using trimmed mean of M values (TMM) in EdgeR. Normalized TMM counts of genes were compared for differential expression between the different conditions using EdgeR’s Quasi-likelihood ratio (glmQLFit). Genes were considered statistically expressed when the absolute fold change ≥2 and false discovery rate (FDR) < 5 %. RNA-seq data supporting the findings was deposited in BioProject. For the *ATRX* IFF lines, the quality of FASTQ files was confirmed using FASTQC (version 0.11.9). Reads were aligned to hg38 using STAR (version 2.7.5b) (–outFilterMultimapNmax 10 –outFilterMismatchNmax 10 –outFilterType BySJout –outFilterIntronMotifs RemoveNoncanonicalUnannotated). BAM files were imported into R using Rsubread featureCounts (1.32.4) and differential expression analysis was performed using DeSEQ2 (version 1.36.0). Genes were considered differentially expressed with a Ilog2FCI ≥0.75 and adj-pvalue of ≤0.05.

For cell type enrichment analysis, xCell [12] was used to estimate the various immune and stroma cell types. Tumors’ *ATRX* mutation status was used to dichotomize each cell type into *ATRX* MUT and *ATRX* WT groups.

### 2.7. In vivo studies

All animal experiments were approved and monitored by The Institute of Cancer Research Animal Welfare and Ethical Review Body in compliance with the guidelines set by the UK Home Office Animals (Scientific Procedures) Act 1986 and the United Kingdom National Cancer Research Institute for welfare of animals in cancer research.

Xenograft studies were carried out in NOD.Cg-Prkdcscid Il2rgtm1Wjl/SzJ (NSG) mice purchased from Charles River. 5 × 10^6^ NBL-S cells were injected subcutaneously into 4 mice per line and monitored twice weekly. Tumor size was determined by caliper measurements and mice were sacrificed when the mean diameter approached tumor size limit of 12 mm.

Transgenic mice were monitored for palpable tumor twice per week for >120 days. Genotyping was performed at weaning by Transnetyx using real-time PCR. C57BL/6 *Atrx*-floxed mice [13] were obtained from the MRC Weatherall Institute of Molecular Medicine (Oxford, UK). C57BL/6 *Atrx*-floxed mice were crossed with *Th-Cre* mice (129SvJ. Cg-Th^tm1(Cre)Te^/KiegICR, purchased from EMMA as B6.129X1 and backcrossed into 129X/SvJ background at ICR). The C57BL/6 *Atrx*--floxed mouse colony was backcrossed into 129X1/SvJ at Charles River.

### 2.8. Patient-derived xenograft (PDX)

PDXs were established from neuroblastoma tumor biopsies obtained at diagnosis or at relapse, as described previously, following informed consent of parents or guardians [14]. This study was performed in accordance with the recommendations of the European Community (2010/63/UE) for the care and use of laboratory animals. Experimental procedures were approved by the ethics committee of the Institut Curie CEEA-IC#118 (Authorization APAFIS#11206–2017090816044613-v2) in compliance with the international guidelines. The establishment of PDX received approval by the Institut Curie institutional review board OBS170323 CPP ref 3272; number dossier 2015-A00464–45. Tumor samples were engrafted subcutaneously in immunocompromised mice. Upon successful establishment (> Passage 2) and reach of ethical size, PDX tumors were cryopreserved for banking, morphological and molecular analyses according to procedures described previously. For histological analysis, tumors were fixed in 4 % paraformaldehyde and embedded in paraffin.

### 2.9. Histopathological analysis and scoring

Neuroblastoma patients with somatic *ATRX* variants (n = 3) were identified from clinical databases. Neuroblastoma patients with *MYCN* amplification (n = 6) and patients with neither *MYCN* amplification nor *ATRX* mutation (n = 13) for the control groups were identified from pathology databases. Archived material from primary diagnostic biopsies was used for assessment to reduce the possibility of confounding by chemotherapy-induced change. Unstained spare slides were pulled and submitted for a panel of immunohistochemistry including CD3, CD20 and CD68kp1 in all cases. *ATRX* variant cases were additionally interrogated using CD14 and CD163. H&E-stained slides and internal controls were used to evaluate immunoreactivity. Representative areas of tumor were chosen by two reviewers and the number of positive cells per high-power field (40× objective) was enumerated for each marker.

### 2.10. Statistical analysis

Statistical analysis was performed with Prism (GraphPad Software Inc, version 10). When comparing two groups, the unpaired Student *t*-test or Mann-Whitney test was used for normally or not normally distributed data, respectively. One-way ANOVA was used to compared more than two groups. The log-rank (Mantel-Cox) test was used to analyse the *P* value of survival curves. Pearson test (normally distributed data) or Spearman r test (not normally distributed data) was used for correlation studies. *P* values < 0.05 were considered significant. Data are shown as mean ± SEM or SD as indicated in the figure legends.

## 3. Results

### 3.1. CRISPR-Cas9-mediated knockout of ATRX induces ALT in NBL-S cell lines

**Fig. 1.**
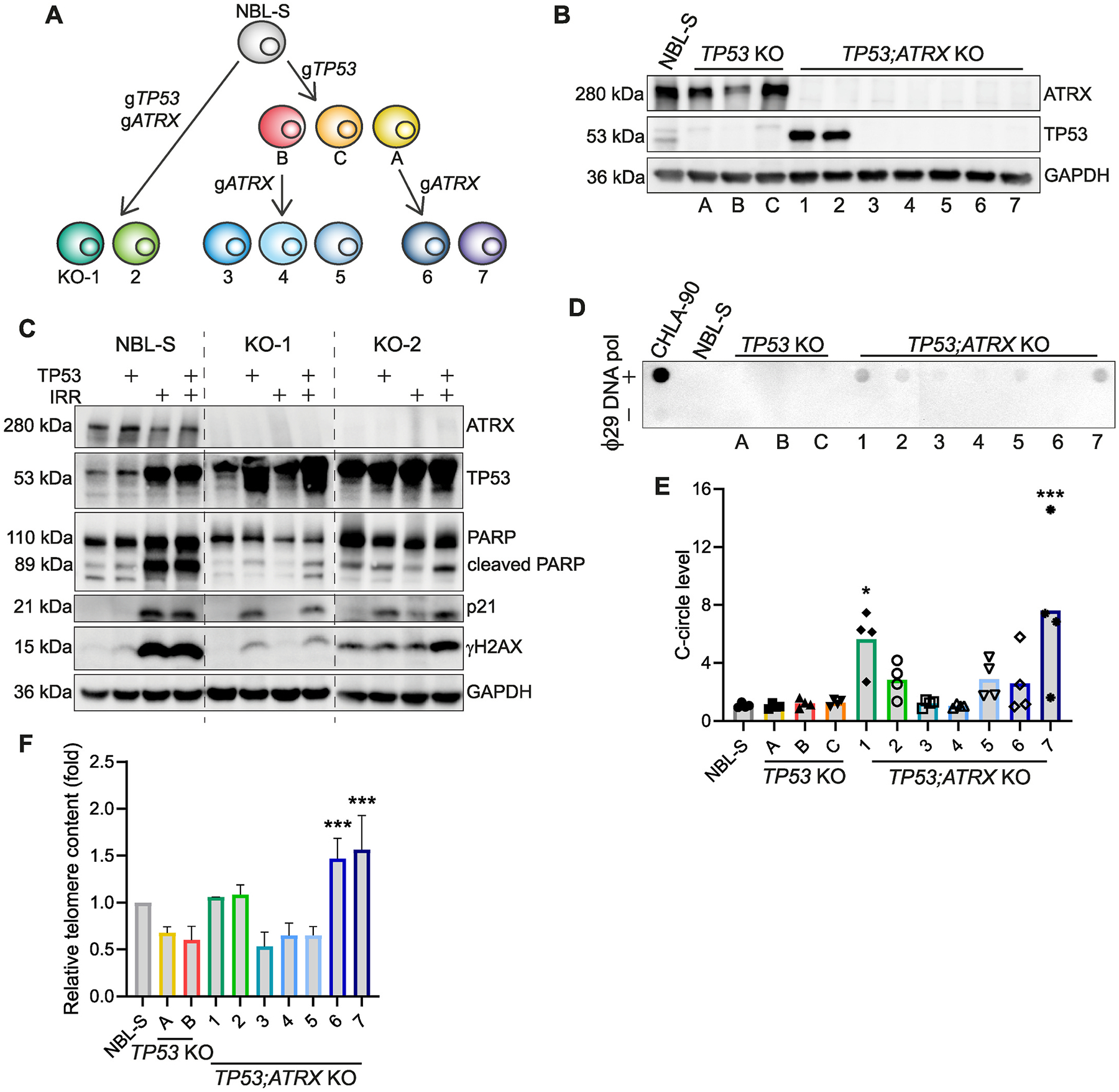
Knockout of *ATRX* induces ALT in NBL-S cell lines. **A,** Schematic of generation of *TP53* knockout (KO) (A, B, C) and *TP53;ATRX* KO (KO-1 to KO-7) NBL-S cell lines by CRISPR-Cas9 technology. **B,** Western blot analysis of expression levels of ATRX and TP53 in NBL-S mutant lines. **C,** Immunoblots of wildtype (WT), KO-1 and KO-2 NBL-S cells following 10 Gy irradiation and/or exogenous expression of TP53 using ATRX, TP53, total PARP, cleaved PARP, p21 and γH2AX antibodies. GAPDH was used as loading control. **D,** Representative dot blot of DNA c-circles amplified by ϕ29 DNA polymerase (alongside no ϕ29 DNA polymerase control) of NBL-S WT and *ATRX* KO cell lines. DNA from the ALT-positive CHLA-90 cell line was used as positive control. **E,** Quantification of dot blots (n = 4 biological replicates). Graph reports individual intensity data points relative to corresponding no ϕ29 DNA polymerase control per each cell line. *P* values, calculated using one-way ANOVA, show the comparison of KO-1 with NBL-S WT and KO-7 with the parental cell line A (*P < 0.05, ***P < 0.001). **F,** Histogram shows relative telomere length by quantitative PCR of NBL-S cell lines. VAV2 was used as the single copy gene to normalize telomere PCR product. The experiment was done in triplicate and in three independent occasions. Data are shown as mean ± SEM, ***P < 0.001. *P* values, calculated using one-way ANOVA, show the comparison of KO-6 and KO-7 with the parental cell line A.

We selected the NBL-S cell line to study the role of ATRX in neuroblastoma, as in keeping with the SKNSH-derived models we have previously generated [9], it is *MYCN* non-amplified, ALT-negative and *TP53* wildtype. We used CRISPR-Cas9 technology to induce frameshift mutations of *ATRX* in NBL-S cells. However, in common with the SKNSH cell line, it did not survive following knockout of *ATRX* in the presence of functional p53 signaling pathway [9]. Thus, CRISPR-Cas9 was used to first inactivate *TP53* and generate three *TP53* knockout cell lines (named A, B and C). A and B cell lines were then transfected to delete *ATRX*. As a result, we generated five *TP53;ATRX* knockout cell lines (Fig. 1A; KO-3-KO-7). By double transfection with both gRNAs targeting *TP53* and *ATRX* we obtained two additional *TP53;ATRX* knockout lines, KO-1 and KO-2 (Fig. 1A). Western blotting analysis confirmed loss of ATRX in all mutant NBL-S lines (Fig. 1B and Supplementary Fig. S1A), however revealed retained expression of *TP53* in KO-1 and KO-2 cell lines (Fig. 1B). To elucidate if TP53 were functional in KO-1 and KO-2 cell lines, we generated TetOn-TP53 inducible versions of WT, KO-1 and KO-2 cell lines, that overexpress wildtype *TP53* upon doxycycline induction. We then triggered DNA damage by γ-irradiation and examined the activation of the TP53 signaling pathway by Western blot. Increased levels of cleaved PARP, γH2AX and the downstream mediator of TP53, p21, were observed in irradiated NBL-S wildtype cells, but not in the KO-1 or KO-2 cell lines (Fig. 1C), suggesting that *TP53* encodes an inactive protein in these cell lines. Moreover, overexpression of *TP5*3 by doxycycline in the KO-1 and KO-2 lines partially rescued the phenotype indicating intact p53 response pathways (Fig. 1C). Interestingly, the expression of *TP53* did not show synthetic lethality with the absence of ATRX. Panel-targeted whole genome sequencing of NBL-S clones further confirmed *TP53* and *ATRX* mutations in the mutant lines (Supplementary Fig. S1B). In conclusion, CRISPR-Cas9 knockout of *ATRX* is only possible when the TP53 signaling pathway is inactive in NBL-S cells, however re-expression of wildtype *TP53* is not sufficient to induce cell death in *ATRX* knockout cells.

*ATRX* mutations are found in 55 % of ALT-positive neuroblastomas [15]. To assess whether deletion of *ATRX* induces ALT activation, we examined the presence of DNA c-circles (which accumulate in ALT-positive cells) in wildtype and mutant NBL-S lines [16]. C-circles were detected at variable levels in almost all *TP53;ATRX* mutant cell lines but not in wildtype or *TP53* knockout cell lines. By quantification of dot blot intensity, we found that *TP53;ATRX* knockout KO-1 and KO-7 cell lines exhibited a significantly higher c-circle signal compared to no polymerase controls, whereas KO-2, KO-5 and KO-6 cell lines showed a trend towards increased c-circles levels (Fig. 1D–E). Quantitative PCR analysis of telomeric regions showed that *TP53;ATRX* knockout KO-6 and KO-7 cell lines have significantly longer telomeres than *ATRX* wildtype *TP53* knockout cell lines (Fig. 1F) [11]. Further analysis showed that knockdown of *ATRX* by shRNA is not sufficient to trigger the ALT pathway in NBL-S cells in a *TP53* wildtype background (Supplementary Figs. S1C–D). Similarly, knockout or knockdown of *ATRX* did not induce an ALT pathway in SKNSH cells (Supplementary Figs. S1D–F). Altogether our results indicate that induction of ALT is cell context dependent, with LoF of ATRX activating c-circle formation in some NBL-S but not in SKNSH-derived, *TP53* knockout cell lines.

### 3.2. Knockout of ATRX associates with upregulation of inflammatory response pathways in NBL-S lines

**Fig. 2.**
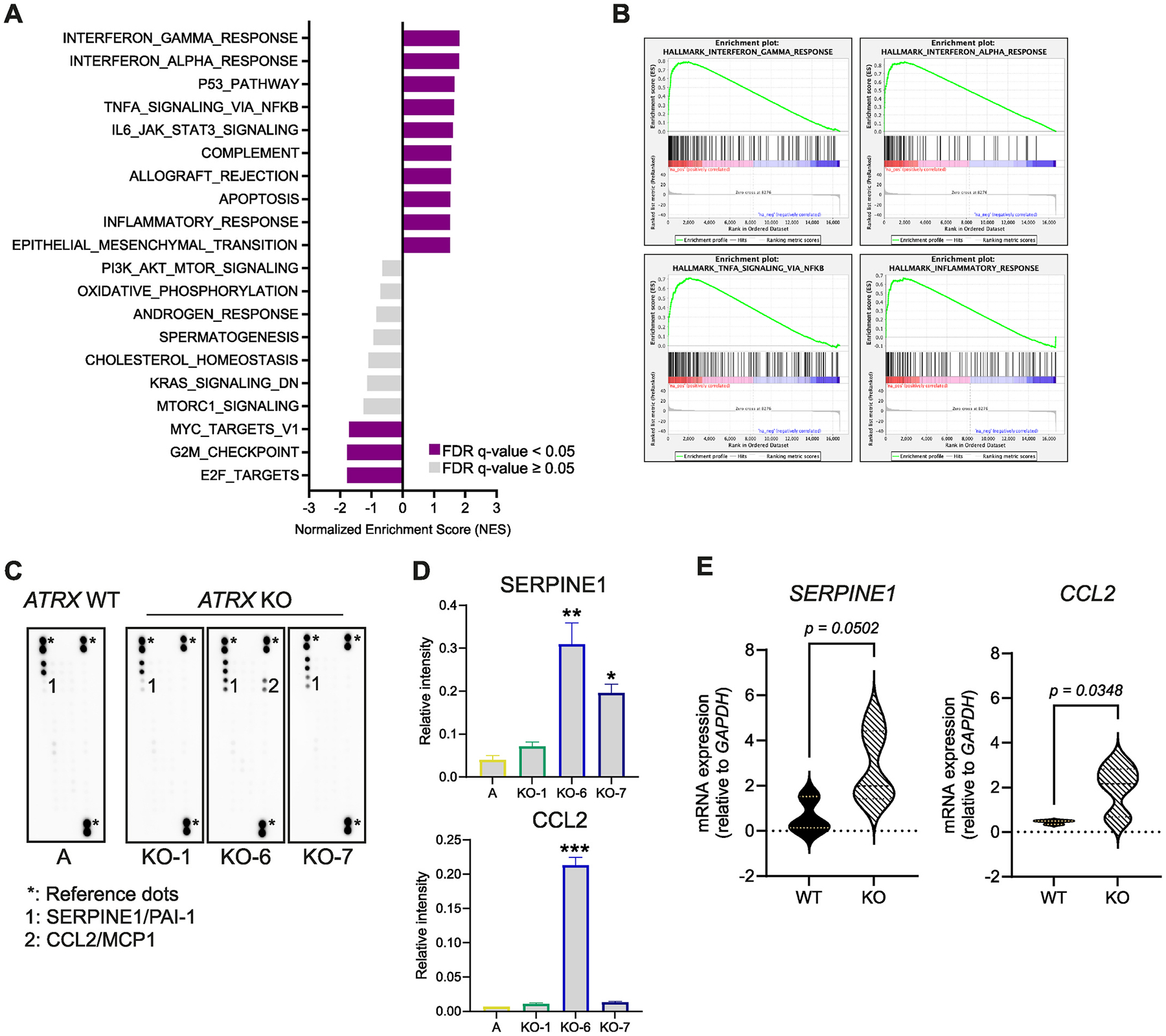
Knockout of *ATRX* associates with upregulation of inflammatory response pathways in NBL-S lines. **A,** List of top ten upregulated and downregulated terms based on annotated deregulated genes and (**B**) enrichment plots of top inflammation-related pathways (pre-ranked) by Gene Set Enrichment Analysis (GSEA) of *ATRX* KO NBL-S cell lines (KO-1, KO-2, KO-6, KO-7) compared to *ATRX* WT lines (NBL-S, A, C). **C,** Dot blots of human cytokine array of *ATRX* WT and *ATRX* KO-1, KO-6 and KO-7 cell line supernatants. **D,** Quantification of chemiluminescent intensity of SERPINE1 and CCL2 normalized to the mean density of the reference dots. One-way ANOVA was used to measure the statistical significance between A and KO-1, KO-2 or KO-7, separately (*P < 0.05, **P < 0.01, ***P < 0.001). **E,** Violin plots of the expression of *SERPINE1* and *CCL2* in NBL-S *ATRX* WT (NBL-S, A, B, C) or KO lines (all 7 KO lines: KO-1 – KO-7) measured by qRT-PCR (normalized to *GAPDH* expression). The experiment was done in triplicate and in three independent occasions. *P* values were measured with unpaired Student *t*-test.

Focusing on KO-1, KO-2, KO-6 and KO-7, which most robustly showed hallmarks of ALT, we investigated transcriptional differences compared to wildtype cells by RNA sequencing. Gene set enrichment analysis (GSEA) showed a significant upregulation of several pathways involved in the inflammatory response including the interferon gamma response, interferon alpha response and TNF-alpha signaling via NF-kB pathways. Also of note, the epithelial-to-mesenchymal transition gene set was enriched in *TP53;ATRX* knockout cells compared with parental cell lines (Fig. 2A–B). RNA sequencing of SKNSH-derived *ATRX* knockout cell lines previously generated [9] also showed significant upregulation of the same inflammatory response pathways (Supplementary Figs. S2A–B). Together this indicates that ATRX LoF is associated with upregulation of genes in inflammatory pathways, but this may not be associated with activation of ALT.

To investigate if the upregulation of inflammatory pathways results in phenotypic changes, we performed a human cytokine array using the culture medium of *TP53* knockout A cells and *TP53;ATRX* knockout KO-1, KO-6 and KO-7 cells. An increased level of serpin family E member 1 (SERPINE1) was identified in the conditioned media (CM) of *ATRX* mutant lines and the chemokine ligand 2 (CCL2) was found only in the CM of KO-6 cells compared to wildtype (Fig. 2C–D). A list of chemokines included in the array and their relative expression in samples is shown in Supplementary Data 1. Quantitative RT-PCR analysis confirmed upregulation of *SERPINE1* and *CCL2* in all seven *ATRX* knockout lines versus wildtype, albeit with high variability across clones of the same genotype (Fig. 2E). Nonetheless, these findings indicate that LoF of ATRX may be associated with production of pro-inflammatory, macrophage-recruiting cytokines, such as SERPINE1 and CCL2.

### 3.3. ATRX mutations mediate macrophage infiltration in xenograft and PDX models

**Fig. 3.**
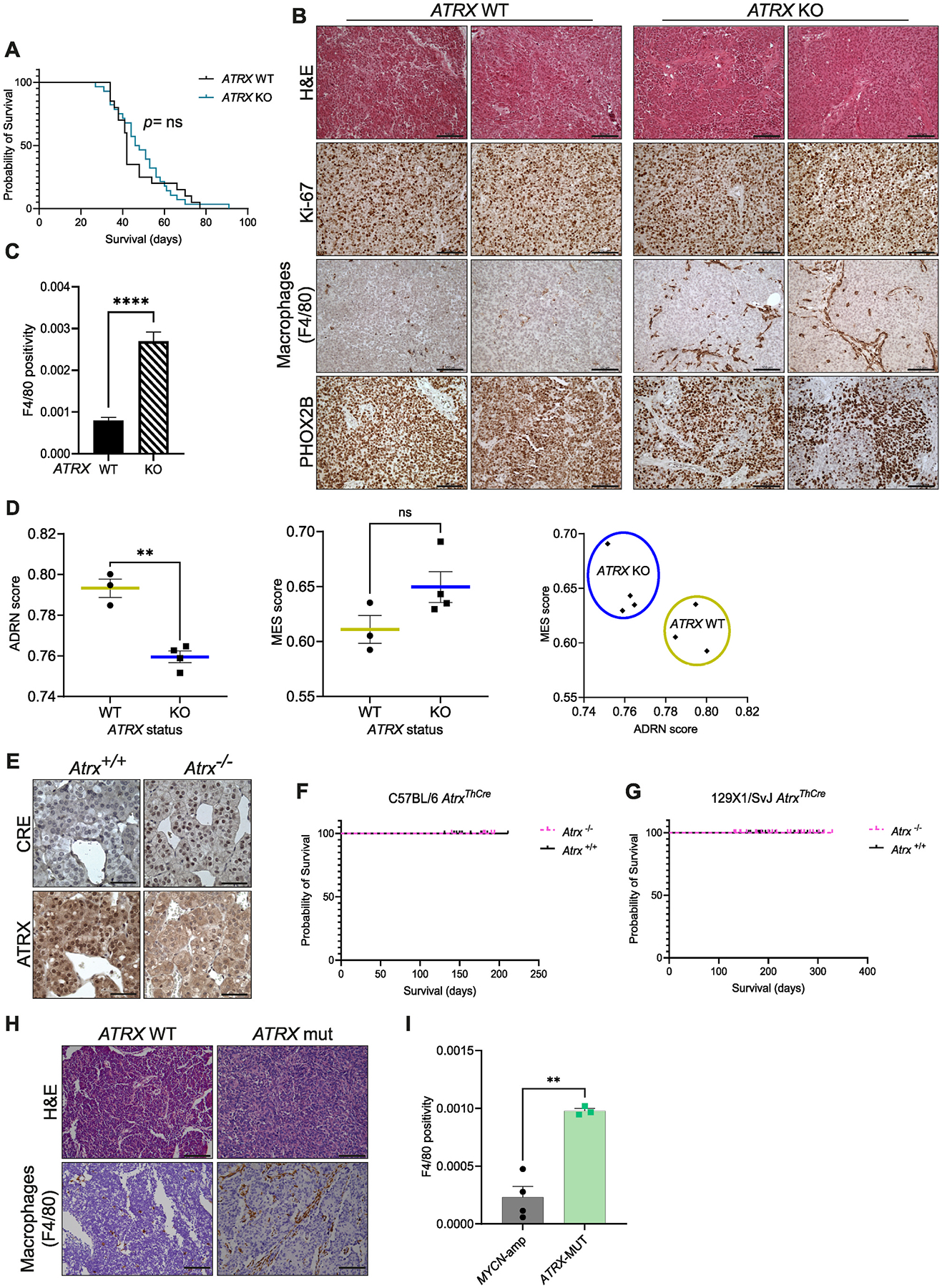
*ATRX* mutations mediate macrophage infiltration in xenograft and PDX models. **A,** Kaplan-Meier analysis of mice bearing subcutaneous xenografts of *ATRX* mutant (KO-1, KO-2, KO-6, KO-7) or wildtype (NBL-S, A and C) NBL-S cells. *P* value calculated with log-rank (Mantel-Cox) test. **B,** Hematoxylin and eosin (H&E) staining and immunohistochemistry analysis for Ki-67, F4/80 and PHOX2B, in two representative *ATRX* WT (A, C) and KO (KO-2, KO-6) xenograft tissues. Scale bars, 100 μm. **C,** Histogram shows quantification of F4/80 (mouse macrophage marker) intensity in *ATRX* WT versus *ATRX* KO slides (n = 6, mean ± SEM, *t*-test). **D,** Adrenergic (ADRN) or mesenchymal (MES) gene signature scores and their correlation in *ATRX* WT (NBL-S, A and C) versus *ATRX* KO (KO-1, KO-2, KO-6, KO-7) NBL-S lines by RNA-seq analysis (*t*-test was used for left and middle panel, **P < 0.01). Right panel, Pearson r correlation. **E,** Representative images of immunohistochemistry analysis of adrenal medullas of *Atrx* wildtype (*Atrx*^+*/*+^) and knockout (*Atrx*^−*/*−^) mice using antibodies against Cre recombinase and ATRX (nuclear staining). Scale bars, 50 μm. **F,** Kaplan-Meier curve of *Th*-driven *Atrx*^−*/*−^ mice (n = 20) and *Atrx*^+*/*+^ mice (n = 30) in the C57BL/6 background (*P* value not significant, log-rank (Mantel-Cox) test). **G,** Kaplan-Meier curve of *Th*-driven *Atrx*^−*/*−^ mice (n = 37) and *Atrx*^+*/*+^ mice (n = 38) in the 129X1/SvJ background (*P* value not significant, log-rank (Mantel-Cox) test). **H,** Representative images of H&E staining and immunohistochemistry analysis of F4/80 of *ATRX* mutant PDXs or MYCN-amplified PDXs as control. Scale bars, 100 μm. **I,** Histogram reports the quantification of F4/80 positive staining of *ATRX* mutant PDXs and the *ATRX* IFF AMC772 patient-derived tumoroid xenograft compared to *MYCN*-amplified PDXs (mean ± SEM, *t*-test, **P < 0.01).

Given the secretion of at least two chemokines known to promote macrophage recruitment in *ATRX* LoF models, we established xenograft models to examine the tumor microenvironment. *ATRX* wildtype or knockout cells were subcutaneously injected in athymic nude Crl:NU (NCr)-*Foxn1*^*nu*^ mice and NOD.Cg-Prkdcscid Il2rgtm1Wjl/SzJ (NSG) mice but engrafted exclusively in the NSG strain. Median latency time of *ATRX* wildtype or knockout xenografts was 42 or 47 days, respectively, with no significant difference (Fig. 3A). This was concordant with immunohistochemistry (IHC) analysis of Ki-67 expression which does not vary between the groups (Fig. 3B). However, there was a markedly higher intensity of the F4/80 macrophage marker in *ATRX* knockout xenografts (Fig. 3B–C). A similar significant increase in F4/80 intensity was found in *TP53;ATRX* knockout SKNSH xenografts, compared to *TP53* knockout SKNSH xenografts (Supplementary Figs. S2C–D).

Xenografts of ALT-negative NBL-S KO-3 and KO-4 cell lines also showed greater recruitment of macrophages than *TP53* knockout *ATRX* wildtype NBL-S xenografts (Supplementary Figs. S2E–F). This further suggests that loss of ATRX rather than the activation of ALT, mediates an inflammatory phenotype.

Interestingly, the expression of the adrenergic marker PHOX2B was reduced in the mutant xenografts compared to wildtype (Fig. 3B, Supplementary Fig. S2E). Based on this and the upregulation of genes implicated in epithelial-to-mesenchymal transition found by GSEA, we measured mesenchymal and adrenergic signature scores from neuroblastoma cell line RNA-seq data [17,18]. This identified a significant decrease of adrenergic score in *ATRX* knockout NBL-S lines compared to their wildtype counterparts (Fig. 3D).

To explore the consequence of *ATRX* LoF within the context of spontaneously growing tumors with an intact host immune system, C57BL/6 *Atrx*-floxed mice [13] were crossed with mice expressing *Cre* recombinase under the control of the tyrosine hydroxylase (*Th*) promoter (Fig. 3E). *Atrx*^*ThCre*^ mice were monitored for at least 4 months and no palpable tumor was detected in *Atrx* knockout mice (Fig. 3F). To ask whether *Atrx*^*ThCre*^ knockout penetrance depends on the mouse strain, we rederived C57BL/6 *Atrx*-floxed mice in the 129X1/SvJ strain, which is commonly utilized to generate neuroblastoma genetically engineered mouse models (GEMMs) [19,20]. *Atrx*^*ThCre*^ mice in the 129X1/SvJ background also did not develop tumors (Fig. 3G). Immunohistochemistry analysis confirmed loss of ATRX protein expression in the adrenal medulla of *Atrx*^*ThCre*^ mice (Fig. 3E). In summary, loss of *Atrx* in *Th*-expressing cells is not sufficient to drive neuroblastoma in mice, suggesting that either *ATRX* mutations may occur at a different timepoint during embryo development and/or additional events are required to drive tumorigenesis.

Next, we analyzed the presence of macrophages in patient-derived xenograft (PDX) models established in NSG mice. We identified one patient-derived tumoroid with an *ATRX* IFF mutation and two PDX samples with single-nucleotide mutations of *ATRX*: one with a missense mutation in the helicase domain (p.(Gly1748Arg)) and one with a stop gain (c.6391C > T) in one allele and a missense mutation in the second allele (p.ser1941Ile) (Supplementary Data 2). We performed IHC analysis for macrophage infiltration in comparison with four *MYCN*-amplified PDXs. *ATRX* mutant PDXs showed greater positivity to F4/80 staining on IHC analysis than the *MYCN*-amplified PDXs (Fig. 3H–I). Altogether our results strongly indicate that multiple types of mutation impacting *ATRX* function may associate with an inflammatory phenotype and infiltration of macrophages in neuroblastoma models.

### 3.4. ATRX IFF alterations are also associated with an inflammatory phenotype in neuroblastoma

**Fig. 4.**
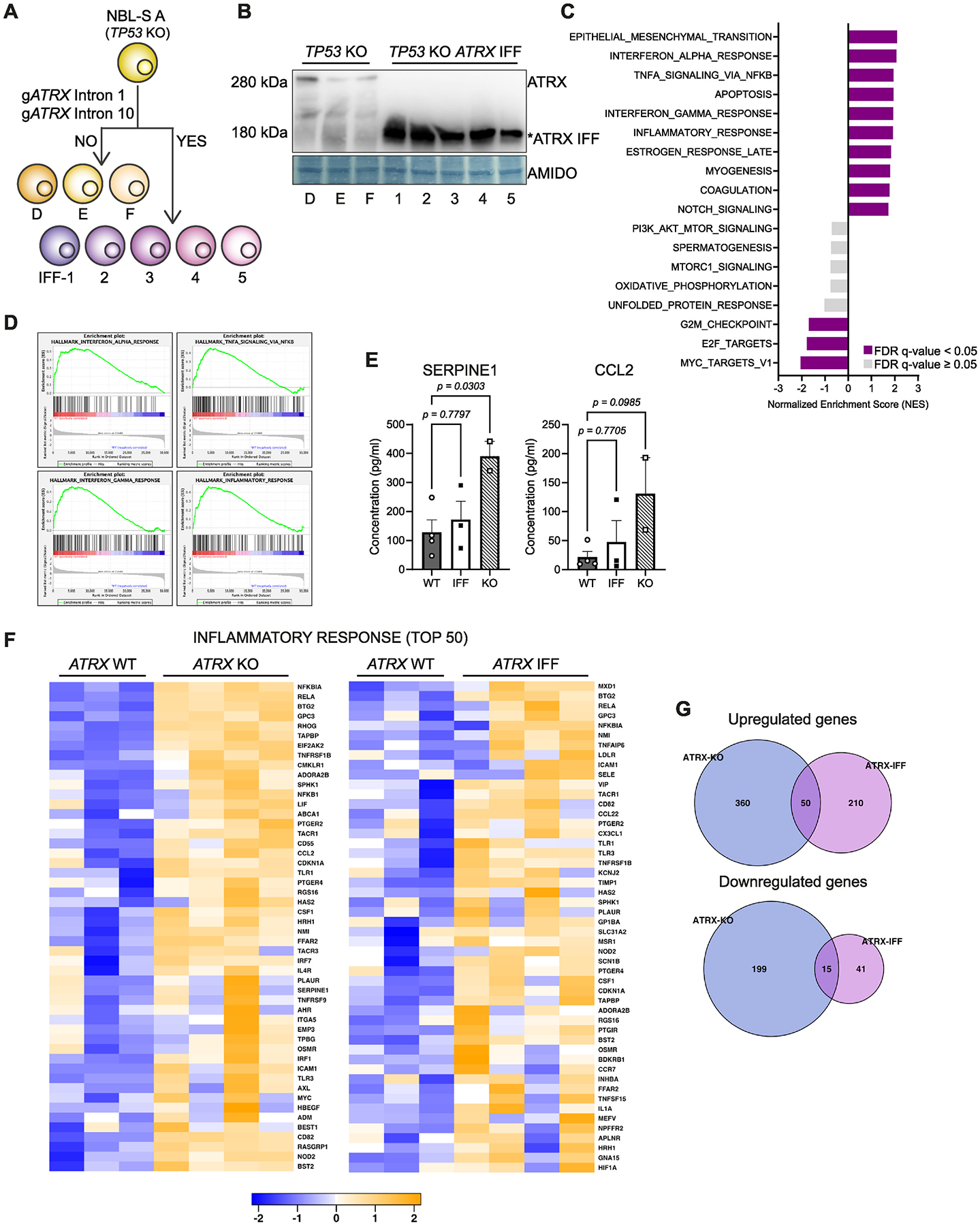
*ATRX* IFF alterations are also associated with an inflammatory phenotype in neuroblastoma. **A,** Schematic of generation of *ATRX* WT (D, E, F lines in yellow gradients, when IFF editing failed) and *ATRX* [1–11] IFF (IFF-1 to IFF-5 lines in purple gradients) isogenic models using NBL-S A cells by CRISPR-Cas9. **B,** Western blot analysis of ATRX in NBL-S lines. Long exposure image shows bands of full-length ATRX in D, E and F cell lines but not in IFF-1, IFF-2, IFF-3, IFF-4 and IFF-5 *ATRX* mutant lines (*saturated bands). **C,** Top ten upregulated and downregulated terms based on annotated deregulated genes and (**D**) enrichment plots of top inflammation-related pathways by GSEA of *ATRX* IFF (IFF-2, IFF-3, IFF-4, IFF-5) vs WT (A, D, E) NBL-S cell lines. **E,** Histograms show SERPINE1 and CCL2 levels in cell culture supernatant of *ATRX* IFF (IFF-1, IFF-2 and IFF-4) or KO (KO-5, KO-7) NBL-S cell lines compared to *ATRX* WT lines (A, B, D and E) (mean ± SEM, *P* values by one-way ANOVA). **F,** Heat maps showing normalized counts of the top 50 upregulated genes in the inflammatory response gene set calculated by GSEA in *ATRX* KO (KO-1, KO-2, KO-6, KO-7) vs WT (NBL-S, A, C) (left) or *ATRX* IFF (IFF-2, IFF-3, IFF-4, IFF-5) vs WT (A, D, E) (right) NBL-S cell lines. **G,** Venn diagrams of the intersection of upregulated (top) or downregulated (bottom) genes between *ATRX* KO (KO-1, KO-2, KO-6, KO-7) and *ATRX* IFF (IFF-2, IFF-3, IFF-4, IFF-5) cell lines (FDR <5 % and absolute ${\text{log}}_{2}^{\text{foldchange}}$ ≥ 0.75).

Next, we asked whether *ATRX* IFF mutations also mediate an inflammatory phenotype in neuroblastoma. Using the NBL-S *TP53* knockout clone A, we used CRISPR-Cas9 to generate an exon 2–10 deletion, causing an exon 1–11 IFF of *ATRX* (Fig. 4A). *ATRX* wildtype clones, where CRISPR-Cas9 failed to generate an IFF, and *ATRX* IFF clones were screened by PCR and then validated by Western blot and targeted sequencing analyses (Fig. 4B). *ATRX* IFF (IFF-2, IFF-3, IFF-4, IFF-5) and wildtype (A, D, E) NBL-S clones were transcriptionally profiled by RNA-seq. GSEA showed significant upregulation of the same inflammatory response pathways and the epithelial-to-mesenchymal transition pathway in keeping with the *ATRX* knockout cell lines (Fig. 4C–D), suggesting that these distinct alterations of *ATRX* in neuroblastoma can both mediate this phenotype.

Cytokine array analysis of the supernatant from *ATRX* wildtype (A, B, D and E), *ATRX* IFF (IFF-1, IFF-2 and IFF-4) and *ATRX* knockout (KO-5 and KO-7) as controls was performed. This confirmed increased levels of SERPINE1 and CCL2 in *ATRX* knockout lines, but no significant difference in the IFF lines, and significant heterogeneity in cytokine expression between individual clones was identified (Fig. 4E and Supplementary Data 3). There was good concordance between GSEA of the inflammatory response pathway genes between *ATRX* knockout and IFF models (Fig. 4F). However, although both *ATRX* knockout and IFF models showed clear similarities in inflammation-associated gene expression, there were a limited number of overlapping genes that reached the threshold for statistical significance across both models (Fig. 4G and Supplementary Data 4).

### 3.5. ATRX mutations mediate an immunogenic phenotype in neuroblastoma patient RNA sequencing datasets

**Fig. 5.**
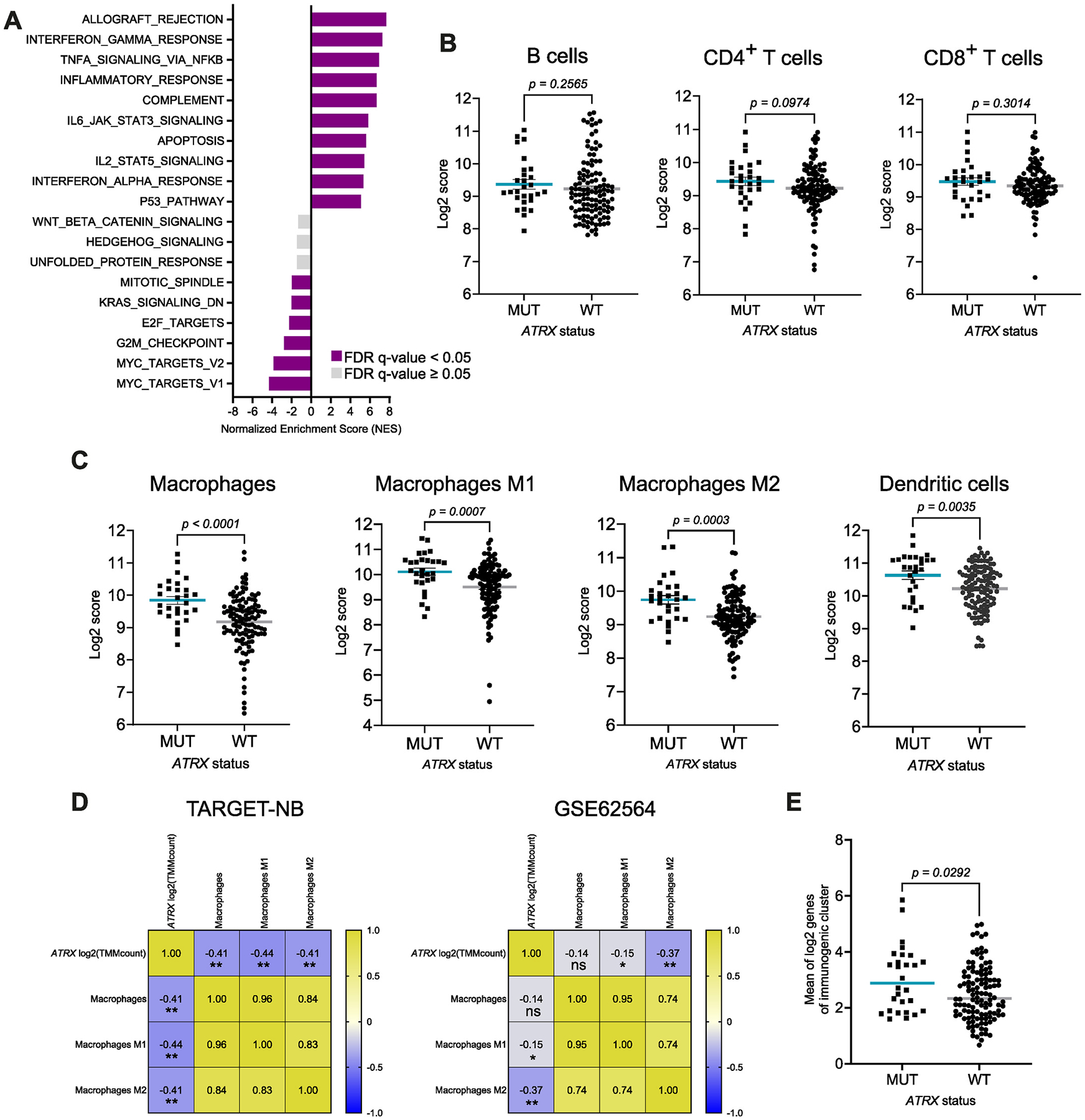
*ATRX* mutations mediate an immunogenic phenotype in neuroblastoma patient RNA sequencing datasets. **A,** Top 10 upregulated and downregulated terms of GSEA of *ATRX* mutant samples in Tumor Neuroblastoma ALT - Westermann −144 - tpm - gencode19 dataset. **B-C,** Plots show lymphoid or myeloid cell type-specific scores of neuroblastoma patient samples in the Westermann dataset based on the *ATRX* status, using the xCell tool. *P* values were calculated with Mann-Whitney test. **D,** Heatmaps indicate the correlation between the expression of *ATRX* and macrophage, macrophage M1 or macrophage M2 scores in TARGET and GSE62564 high-risk neuroblastoma patient datasets (Spearman correlation). **E,** Mean expression of genes that define the immunogenic cluster of neuroblastoma in *ATRX* mutant versus wildtype samples in the Westermann dataset (*P* value calculated with Mann-Whitney test).

To corroborate our *in vitro* and *in vivo* results with neuroblastoma patient datasets, we interrogated the dataset from Hartlieb et al. [15] (Tumor Neuroblastoma ALT - Westermann – 144 - tpm - gencode19, in R2 database), where 144 primary and relapsed neuroblastomas were examined for *ATRX* status. Interestingly, GSEA of patient samples revealed upregulation of inflammatory response-related pathways in *ATRX*-mutated neuroblastomas (Fig. 5A), in line with our results. Next, we used xCell tool (gene signature-based method) [12] to investigate the tumor immune microenvironment of *ATRX*-mutated neuroblastomas in the same patient cohort. Although the scores related to B and T cells were similar in *ATRX* mutant and wildtype samples (Fig. 5B), we found a significantly higher score for macrophages, both M1 and M2, and dendritic cells in *ATRX* mutant patients than their respective counterparts (Fig. 5C). The analysis of two additional available datasets of neuroblastoma patients -TARGET-NB and GSE62564.Fischer – consistently showed a negative correlation between *ATRX* expression and macrophage scores (Fig. 5D).

Sengupta et al. [18] described a 3-cluster classification of neuroblastomas based on their transcriptional profiles. One of the clusters unites the so-called immunogenic tumors that show increased expression in genes involved in the immune response. We, therefore, analyzed the Westermann dataset according to expression of immunogenic genes as per Sengupta et al. and found that *ATRX* mutant neuroblastomas express higher levels of the genes defining the immunogenic cluster when compared to patients with *ATRX* wildtype disease (Fig. 5E, Supplementary Fig. S2G).

In conclusion, these results consistently indicate that *ATRX* mutations associate with higher expression of genes related to an inflammatory immune response and infiltration of cells of myeloid lineage in neuroblastoma patient datasets.

### 3.6. Increase immune infiltration is observed in ATRX IFF neuroblastoma compared with MYCN amplified disease

**Fig. 6.**
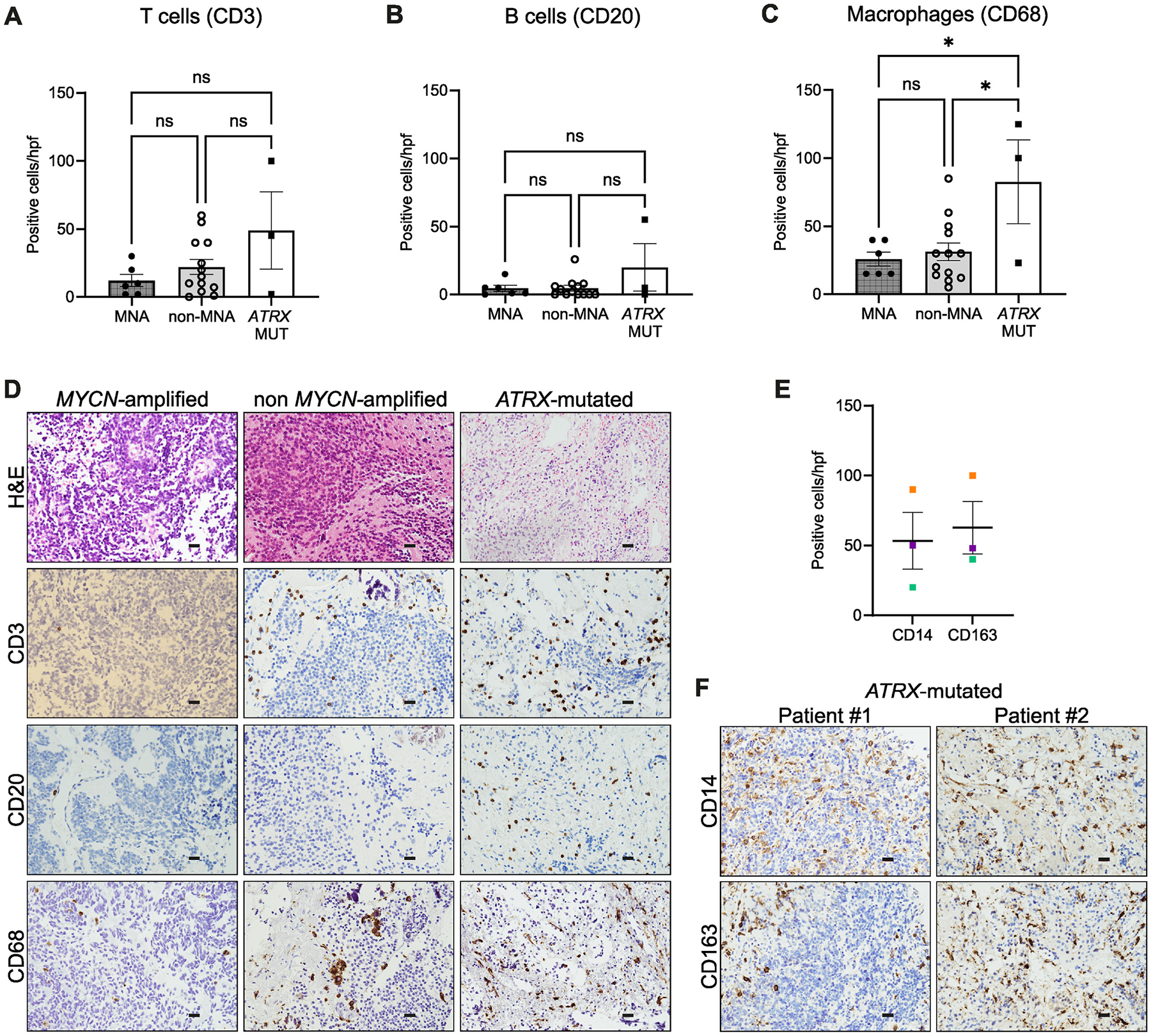
Increased immune infiltration is observed in *ATRX* IFF neuroblastoma compared with *MYCN*-amplified disease. **A, B, C,** Histograms show the comparison of CD3, CD20 or CD68 positive cells between *MYCN*-amplified (MNA), non-MNA and *ATRX* IFF patient samples by histopathological analysis. Data are shown as mean ± SEM (one-way ANOVA, *P < 0.05). **D,** Representative images of H&E and immunohistochemical studies of *ATRX* mutant patients compared to *MYCN*-amplified and non *MYCN*-amplified patients examining dispersed T (CD3) and B (CD20) lymphocytes and macrophages (CD68). Scale bars (black), 20 μm. **E,** Histogram reports number of CD14 and CD163 positive cells in *ATRX* mutant patient samples. Each patient sample is color-coded. **F,** Representative images of immunohistochemistry analysis of CD14 or CD163-positive macrophages in *ATRX* mutant patients. Scale bars (black), 20 μm.

Given the limitations of immunocompromised models to more precisely characterize the nature of the immune infiltrates associated with *ATRX* alterations, we next identified a cohort of FFPE patient samples for more detailed IHC analysis, including 3 samples with *ATRX* IFF’s (3–10, 2–10 and 2–14 multi-exon deletions), 13 non *MYCN*-amplified and 6 MYCN-amplified patient samples. A non-significant trend toward increased CD3 (T cell) and CD20 (B cell) expression was identified in the *ATRX* IFF samples (Fig. 6A–B, D). Consistent with the *in vivo* models and patient datasets, a significant increase in CD68 positive macrophages was identified in *ATRX* mutant neuroblastomas compared with *MYCN*-amplified or non *MYCN*-amplified disease (Fig. 6C–D). We also stained *ATRX* mutant samples with the clinically validated macrophage markers, CD14 (immature monocytes, macrophages and dendritic cells) and CD163 (M2 marker) for further validation, confirming infiltration of cells of myeloid lineage (Fig. 6E–F).

Taken altogether, our results in *MYCN*-amplified neuroblastoma are consistent with others, showing a relatively cold tumor microenvironment [18]. Additionally, in a limited cohort of available *ATRX*-altered neuroblastoma patient samples, we confirm findings from *in vivo* models and patient datasets showing a significant increase in macrophage infiltration.

## 4. Discussion

In recent years it has become apparent that in neuroblastoma *ATRX* mutations define a distinct patient subgroup that are mutually exclusive with *MYCN* amplification, associated with older age at diagnosis and a chronic progressive disease course [4,8]. Despite these differences, all patients diagnosed with high-risk neuroblastoma are currently treated uniformly. Data from large co-operative clinical trials show that survival of patients with *MYCN*-amplified neuroblastoma has improved with intensification of front-line treatment regimens [5]. Although *ATRX* status has not been routinely evaluated in these clinical trials, there is good evidence that older patients continue to do dismally despite intensification of therapy [5,21]. Taken together, this highlights the differences in response to current treatment protocols between subgroups and identifies the urgent need to find alternative approaches to treat patients with *ATRX* mutant neuroblastoma.

A significant challenge in the field is the lack of available models of *ATRX* mutant neuroblastoma. To address this, we have used CRISPR-Cas9 to generate neuroblastoma cell lines with *ATRX* mutations, however in keeping with others [22], we have only been able to achieve this in *TP53* mutant cell lines. In contrast, in patient datasets *ATRX* mutant neuroblastoma is often *TP53* wildtype [23]. Moreover, the cell line observation is also inconsistent with our data in murine models where *Th-Cre* induced *ATRX* deletion in *TP53* wildtype developing neural crest is tolerated. There are a number of potential explanations for this: one possibility is that *ATRX* LoF is only tolerated at specific developmental timepoints in *TP53* wildtype cells, due to tight developmental-stage specific regulation of *TP53* expression during neural crest differentiation [24,25]. Another possibility is that *TP53* is inactivated by alternative means in *ATRX*-mutant neuroblastoma. Alternatively, it may be that the CRISPR-Cas9 process itself, rather than the ATRX loss, results in p53 activation and cell death [26]. The failure of *Th-Cre* mediated deletion of *Atrx* to drive murine neuroblastoma is consistent with experiments in the *Th-ALK* models [19] and it is likely that additional oncogenic drivers will be required to generate murine *ATRX* mutant neuroblastoma models.

Despite the challenges in the generation of *ATRX* mutant neuroblastoma models, our experiments in genetically engineered neuroblastoma cell lines identify cell-intrinsic changes in gene expression due to *ATRX* mutations that result in an inflammatory phenotype and macrophage recruitment. We confirm these findings in a range of patient-derived xenograft models, patient datasets, and patient samples. Our data indicates that cells of a myeloid rather than lymphoid lineage are particularly enriched in *ATRX* mutant neuroblastomas. Furthermore, we identified a general macrophage infiltration with no preponderance of either M1 or M2 macrophages. This is in keeping with a chronic inflammatory phenotype. It is well established that cytokines released during inflammation result in cellular senescence [27], however the induction of senescence also requires intact *ATRX* function [28]. This provides a potential explanation for how *ATRX* mutant cells can induce an inflammatory phenotype but evade immune-mediated surveillance.

In keeping with others [22], we identified a limited number of common genes that were upregulated or downregulated after inducing the same mutation in different cell lines or after inducing different types of *ATRX* alteration in the same cell line. Given the extensive wide-ranging functions of ATRX, and the fact that specific ATRX functions are cell-context dependent [29], this is perhaps unsurprising. However, the upregulation of inflammation-associated pathways and macrophage infiltration was common across all models and datasets that we evaluated.

Alongside an inflammatory phenotype, we also show that in neuroblastoma models both *ATRX* LoF and *ATRX* IFF’s are associated with upregulation of genes associated with epithelial-to-mesenchymal transition. The combination of these findings is in line with previous work demonstrating that neuroblastomas with a mesenchymal signature have a denser infiltration of inflammatory immune cells than adrenergic neuroblastomas [18] and thus we identify *ATRX* alterations as a probable driver of this previously described phenotype. In preclinical models, cells with the immunogenic/mesenchymal phenotype have been shown to be more sensitive to immune checkpoint blockade [18].

Interestingly, clinical trials in other cancers have also identified that *ATRX* mutations may be a predictive biomarker of immune checkpoint inhibitor sensitivity [30,31]. However, it is currently unclear if *ATRX* mutations and/or the immunogenic phenotype are associated with sensitivity to immunotherapies commonly used in children with neuroblastoma including anti-GD2 antibody, which is currently given as standard of care. Of note, our studies have not distinguished whether the enhanced myeloid population is more inflammatory (M1-like) or more regulatory (M2-like) nor the relevance of less differentiated regulatory myeloid populations such as myeloid-derived suppressor cells. Since myeloid cells are increasingly recognized as key effectors for both antibody and CAR-T mechanisms of action in solid cancers, future studies will need to characterize the effector phenotype of the infiltrating myeloid cells in *ATRX*-mutated neuroblastoma. Given our findings and the increasing role of immunotherapy in the management of high-risk neuroblastoma, it is imperative that ongoing and future clinical trials of both standard of care and novel immunotherapy agents evaluate whether *ATRX* status is a biomarker of clinical response to these agents.

In conclusion, our findings add to the increasing evidence that *ATRX* mutant neuroblastoma represents a biologically distinct subgroup of patients. The immune microenvironment changes as a result of *ATRX* mutations are likely to be a contributing factor to this recognizable phenotype.

## Supplementary Material

Data4

Tables

Data1

Figures

Data2

Data3

## Glossary

ALT: Alternative lengthening of telomeres
ATRX: Alpha-thalassemia mental retardation X-linked
CCL2: Chemokine ligand 2
CM: Conditioned medium
FDR: False discovery rate
GEMM: Genetically engineered mouse model
GSEA: Gene set enrichment analysis
IFF: In-frame fusion
IHC: Immunohistochemistry
LoF: Loss of function
MNA: *MYCN*-amplified
PDX: Patient-derived xenograft
RT: Room temperature
SERPINE1: Serpin family E member 1
TH: Tyrosine hydroxylase
TMM: Timmed mean of M values
