# Supplementary material for: ATRX mutations mediate an immunogenic phenotype and macrophage infiltration in neuroblastoma
Source: Cancer Lett. Author manuscript; Available in PMC 2025 May 7. (PMC12057689; doi:10.1016/j.canlet.2025.217495)
Supplement: Tables [file NIHMS2078014-supplement-Tables.docx]

**Supplmentary tables.**

**Table S1. List of guide RNAs**

| **Gene** | **Target Site** | **Sequence** | **Generated by** |
| --- | --- | --- | --- |
| *ATRX* | Intron 1 | GGTGGCTCATAATAGAGCAT | Maged Zeineldin |
| *ATRX* | Intron 10 | AGATATACTTAAGTAAGGGG | Maged Zeineldin |
| *ATRX* | Exon 29 | CATCACTCTGACATCTACCA | Sally George |
| *TP53* | Exon 20 | GATGGCCATGGCGCGGACGC | Sally George |

**Table S2. Genotyping Primers**

| **Target Site** | **Sequence** |
| --- | --- |
| ac.outsideATRXG6.F | TGCCCATGATGACACAGACA |
| ac.outsideATRXG11.R | TGACTGTTGCCTTGGCTAGA |
| ac.spanATRXG6.R | GGTAACAACAGACATCTCCTCT |
| ac.spanATRXG11.F | TGCAAGGAAGTCATGAAGCT |

**Supplementary Table S3. Antibodies**

| **Primary Antibodies** | | | |
| --- | --- | --- | --- |
| ATRX | Sigma, HPA001906 | WB 1:1000, IHC 1:200 | RRID:AB_1078249 |
| ATRX | Abcam, ab97508 | WB 1:1000 | RRID:AB_10680289 |
| CRE | Cell signaling, 12830 | IHC 1:200 | RRID:AB_2631055 |
| CD3 | Leica, LN10 | Ready to use | RRID:AB_3073619 |
| CD14 | Cell Marque, EPR3653 | Ready to use | RRID:AB_2827391 |
| CD20 | Dako, CD20CY L26 | 1:500 | RRID:AB_2282030 |
| CD68kp1 | Dako, CD68 KP1 | 1:200 | RRID:AB_578703 |
| CD163 | Cell Marque, MRQ-23 | Ready to use | RRID:AB_1159128 |
| F4/80 | Cell signaling, 70076 | IHC 1:200 | RRID:AB_2799771 |
| GAPDH | Cell signaling, 5174 | WB 1:1000 | RRID:AB_10622025 |
| γH2AX | Cell signaling, 9718 | WB 1:1000 | RRID:AB_2118009 |
| Ki-67 | BD Biosciences, 556003 | IHC 1:500 | RRID:AB_396287 |
| p21 | Cell signaling, 2946 | WB 1:1000 | RRID:AB_2260325 |
| p53 | R&D, HAF1355 | WB 1:2500 | RRID:AB_2303928 |
| PHOX2B | Abcam, ab183741 | IHC 1:500 | RRID:AB_2857845 |

**Supplementary Table S4: Deposited Data**

| RNA-seq: *ATRX*-knockout NBL-S lines | PRJNA1194390 |
| --- | --- |
| RNA-seq: *ATRX-*IFF NBL-S lines | GSE277158 |
