# Supplementary material for: ATRX mutations mediate an immunogenic phenotype and macrophage infiltration in neuroblastoma
Source: Cancer Lett. Author manuscript; Available in PMC 2025 May 7. (PMC12057689; doi:10.1016/j.canlet.2025.217495)
Supplement: Figures [file NIHMS2078014-supplement-Figures.docx]

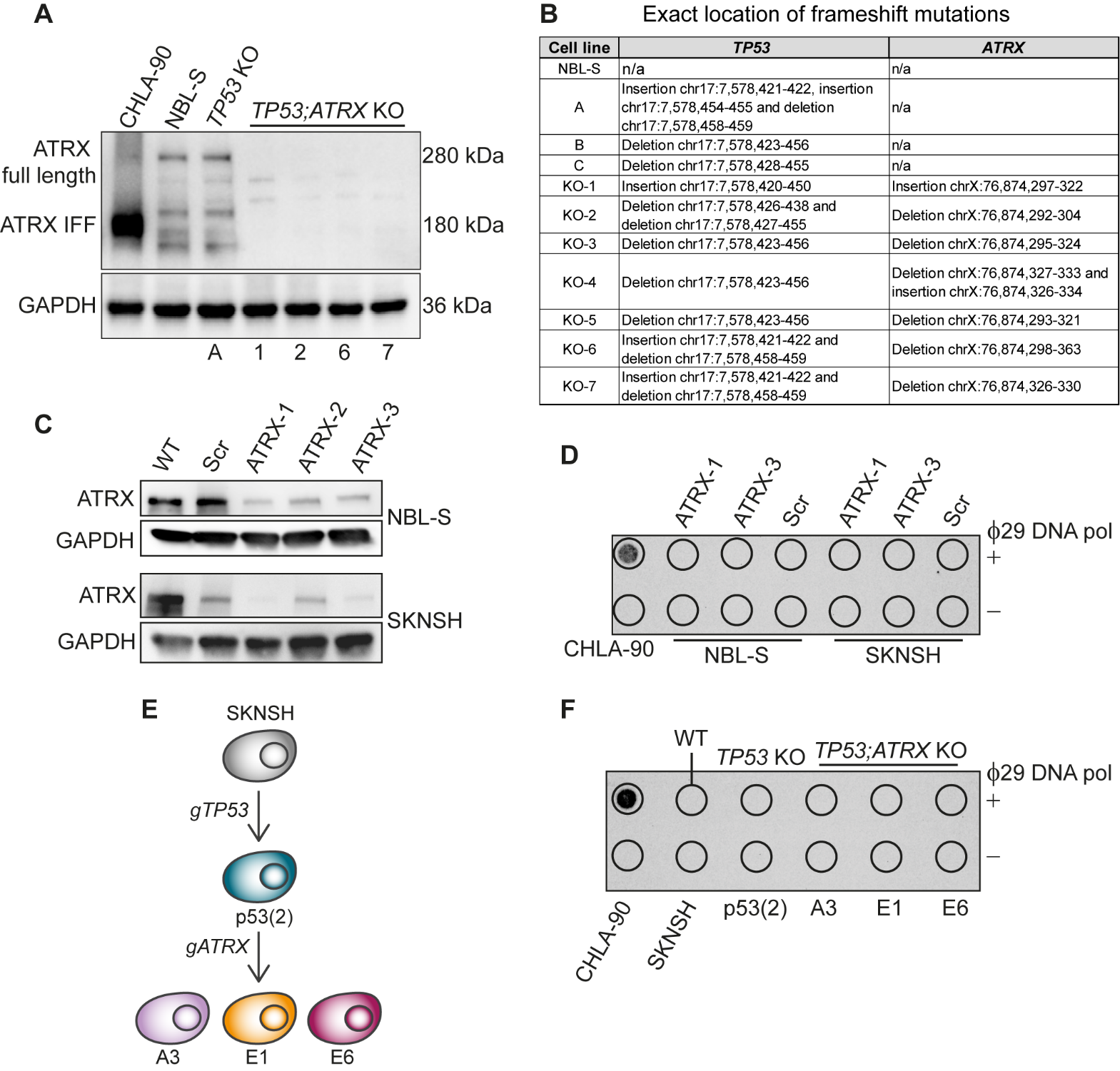


**Supplementary Fig. S1 ALT activation is not induced by *ATRX* downregulation in NBL-S or SKNSH cells.**

**A,** Western blot analysis of expression levels of ATRX in NBL-S KO clones. CHLA-90 cells were used as control for ATRX IFF. GAPDH was detected as loading control. **B,** Exact location of CRISPR-induced frameshift mutations of *TP53* or *ATRX* in NBL-S cell lines by panel-targeted WGS. **C,** Western blot analysis to confirm downregulation of *ATRX* by three shRNAs (ATRX-1, 2 or 3) compared to scrambled shRNA (scr) in NBL-S or SKNSH neuroblastoma lines. GAPDH was detected as loading control. **D,** C-circle assay of NBL-S or SKNSH neuroblastoma cells following downregulation of *ATRX* by two separate shRNA constructs (ATRX-1 and ATRX-3) compared to scrambled shRNA (scr). CHLA-90 cells were used as ALT-positive control. **E,** Schematic of the strategy used to knockout *ATRX* in SKNSH cell line by Cas9. First, p53(2) cell line was obtained by deleting *TP53* and then *ATRX* was knocked-out to establish three *ATRX* mutant lines – A3, E1 and E6. **F,** C-circle analysis in SKNSH cell panel. CHLA-90 was used as ALT-positive control.


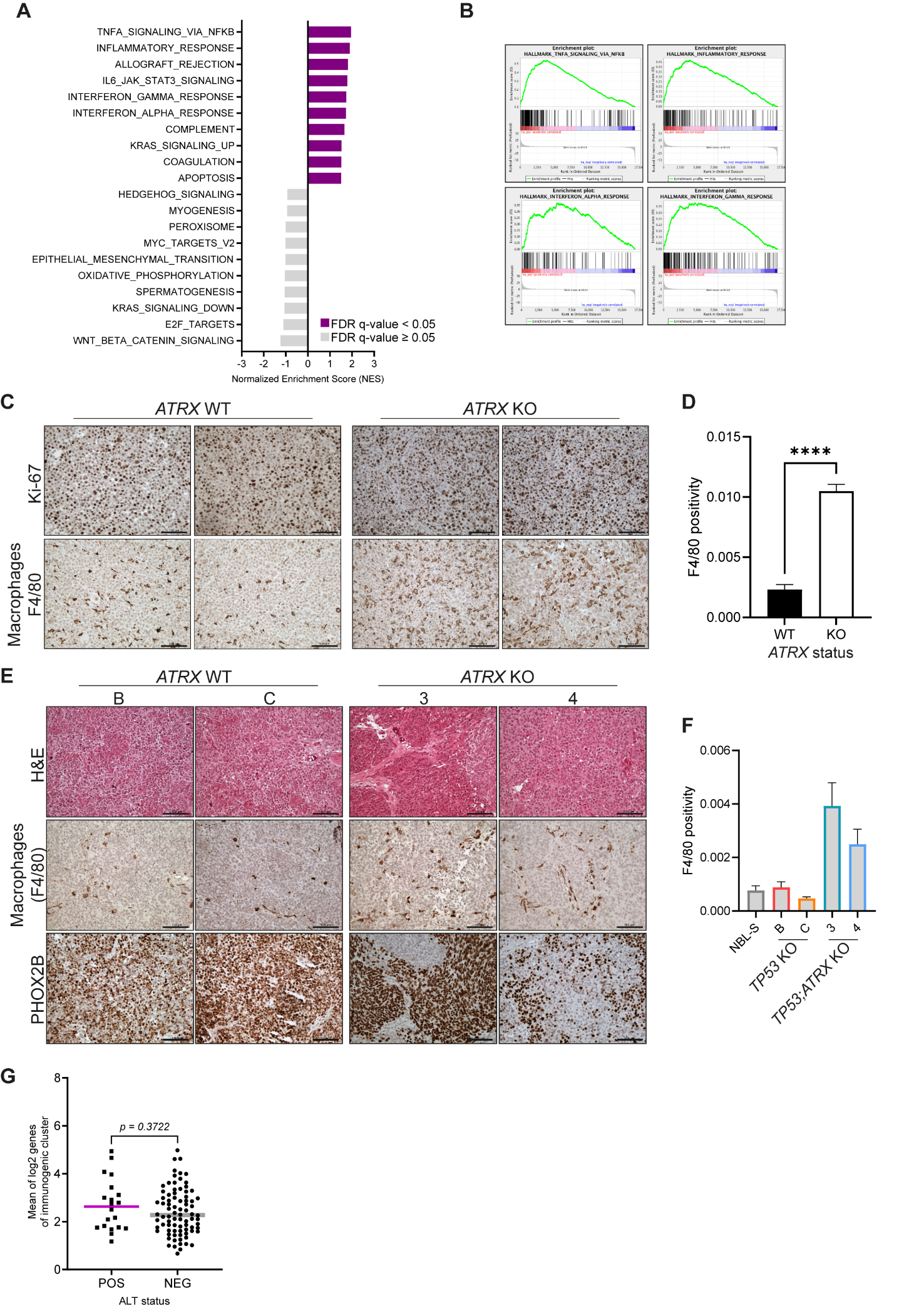


**Supplementary Fig. S2 *ATRX* loss of function associates with increased infiltration of macrophages in xenografts of SKNSH cell lines and ALT-negative *ATRX* KO NBL-S lines.**

**A,** List of the top ten upregulated or downregulated pathways and (**B**) enrichment plots of inflammation-related pathways by GSEA of *ATRX* KO SKNSH lines versus *TP53* KO cells. **C,** Representative images of immunohistochemistry analysis using Ki-67 and F4/80 antibodies of xenografts of *TP53* KO (p53(2), *ATRX* WT) or *TP53;ATRX* KO (E6) SKNSH cell lines. Scale bars, 100 μm. **D,** Histogram shows quantification of F4/80 staining intensity measured by ImageJ. Data are shown as mean ± SEM (n=6, Student *t* test, **P < 0.05, **P < 0.01, ***P < 0.001*). **E**, H&E staining and immunohistochemistry analysis for F4/80 and PHOX2B in xenografts of *ATRX* WT (B, C) and KO (ALT negative KO-3, KO-4) NBL-S cell lines. Scale bars, 100 μm. **F,** Quantification of F4/80 intensity in *ATRX* KO (ALT-negative KO-3, KO-4) versus *ATRX* WT (B, C) slides (n=6, mean ± SEM, one-way ANOVA, **P < 0.05, ***P < 0.001*). For statistical analysis, each *ATRX* KO cell line was compared to the parental cell line TP53 KO B. **G**, The graph shows the mean of the expression of genes that define the immunogenic cluster of neuroblastoma in *ATRX* wildtype ALT positive versus ALT negative samples in the Westermann dataset. *P* value calculated with Mann-Whitney test.
